# Prolapsed ureterocele mimicking a vulval mass in young female with complete duplex system and ureteral stone: A case report

**DOI:** 10.1016/j.ijscr.2023.108087

**Published:** 2023-03-31

**Authors:** Lalu Muhammad Editia Subihardi, Ilham Akbar Rahman, Niwanda Yogiswara, Fikri Rizaldi, Tarmono

**Affiliations:** a Department of Urology, Faculty of Medicine, Universitas Airlangga, Dr. Soetomo General-Academic Hospital, Surabaya, East Java, Indonesia; b Department of Urology, Faculty of Medicine, Universitas Airlangga, Universitas Airlangga Teaching Hospital, Surabaya, East Java, Indonesia

**Keywords:** Ureterocele, Prolapsed Ureterocele, Duplex system, Ureteral stone, Endoscopic resection

## Abstract

**Introduction and importance:**

Ureterocele is a congenital malformation of the ureter with dilatation in the distal part of the ureter. In most cases, this condition was present in childhood. In cases involving adults, this condition is associated with prolapse as well as the formation of stones. Prolapsed ureterocele with stone is considered to be a very rare case. We report a complex case of prolapsed ureterocele in a young female with a protruding mass in the vagina with complete pyeloureteral duplication and stone in the left ureterovesical junction.

**Case presentation:**

A 19-year-old female presented to the hospital with a complaint of protruding mass in the vagina. A Computed Tomography (CT) scan and Intravenous Urography (IVU) confirmed the presence of a left ureterocele with a complete duplex system and stone in the ureterovesical junction. An endoscopic resection of the ureterocele was performed. One year following surgery, the patient was asymptomatic without deterioration of renal function or urinary tract infection.

**Discussion:**

Prolapsed ureterocele in adulthood mimicking the clinical appearance of vulvar mass is considered a very rare case. The imaging examination in this case can be identified through CT-Scan. Surgical treatment of ureterocele consisted of incision, multiple punctures, unroofing, or resection. Considering the complex presentation in this case, we decided to undergo endoscopic resection to prevent the incidence of re-prolapsed which later required a second procedure.

**Conclusion:**

In cases of prolapsed ureterocele associated with urethral stones, endoscopic treatment is a viable option for reducing the risk of recurrent ureterocele prolapse.

## Highlights

• Prolapse ureterocele may present in adult female as vulvar mass.
• The size, shape and location of the ureterocele could be identified using a CT scan.
• Endoscopic incisions are currently the initial procedures for ureterocele management.

## 1 Introduction

Ureterocele is a ureter congenital anomaly where the distal ureter widens at its opening into the bladder forming a sac-like pouch [1]. Ureterocele is one of the disorders that is associated with complete kidney duplication (95 %) [2]. Ureterocele occurs in about 1 out of every 4000 children and mostly occurs in Caucasians. The incidence in women is 4–7 times more often than in men [3]. Most ureterocele cases are diagnosed in children younger than 3 years, but ureterocele can also be found in older children and young adults [4]. Generally, ureterocele in children is found in routine prenatal screening. Adult ureterocele can also be found accidentally during imaging studies, and most commonly presented with no symptoms [5]. Herewith, we discussed a case of prolapsed ureterocele in a young female with a vulval mass along with complete pyeloureter duplication and left vesicoureteral junction (UVJ) stone. This case has been reported in line with the SCARE 2020 criteria [6].

## 2 Presentation of case

**Fig. 1:**
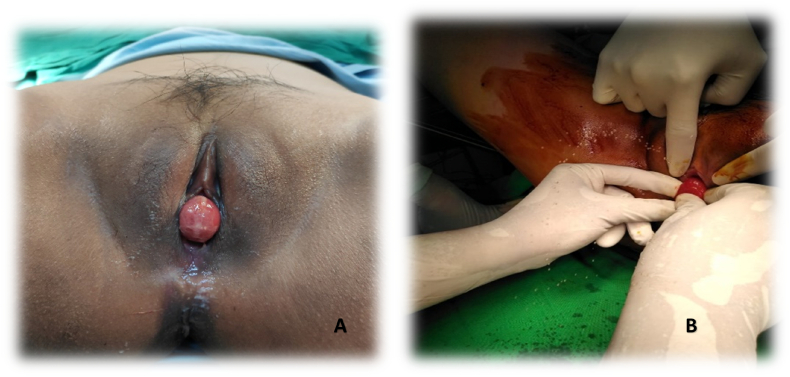
(A). Prolapsed mass from the vulva. (B) Intraoperative surgery is done by Gynecologist: Prolapsed mass released discharge during repositioning (appeared to be urine).

A 19 years old female presented to the hospital with a complaint of a mass arising from the genital organ 3 months ago with urinary retention. The mass may retract spontaneously when the patient lies down on her back, but over time the mass has to be pushed back using a hand to put the mass back in. This mass usually came out while urinating or straining. The patient had difficulty urinating and felt pain when the mass came out. A 5 × 5 cm mass was found on physical examinations that originated from the upper vulva with solid and elastic consistency, and non-tender. The mass seemed to originate from the urethra, and the hymen was intact (Fig. 1). The patient had her first surgery before the hospital submission. The gynecology department performed the surgery, which involved repositioning the mass in the urethra and inserting a urinary catheter.

**Fig. 2:**
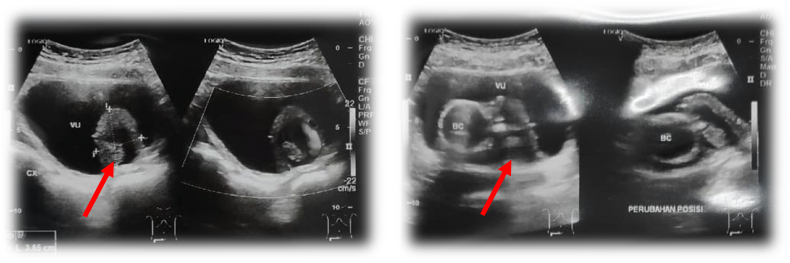
Abdominal ultrasound in patients showing hetero-echoic lesions contained dominant solid mixed cystic with calcification component in it, the size of the mass was 3.6 × 3.8 × 3.1 in the posteroinferior bladder wall (shown by red arrow). (For interpretation of the references to colour in this figure legend, the reader is referred to the web version of this article.)

**Fig. 3:**
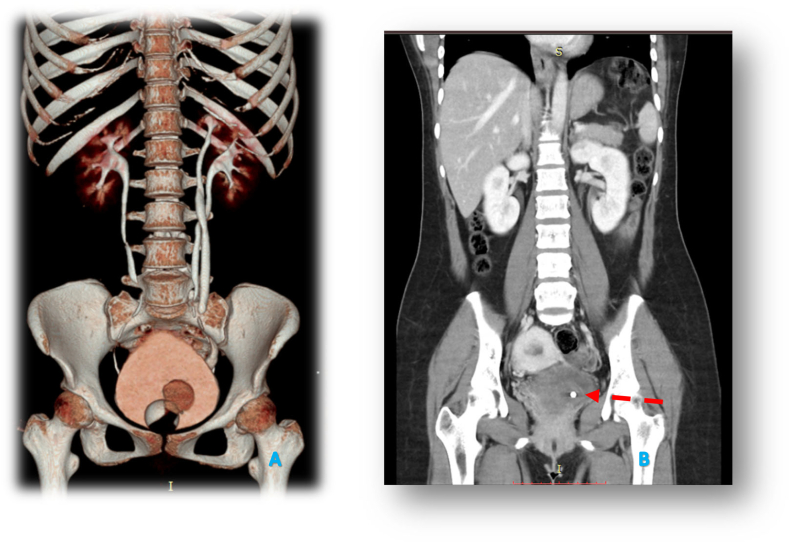
A and B. CT IVU in patients showing the imaging of left intravesical ureterocele left the complete double system and radiopaque shadow on the left UVJ with the impression of stone (shown by red arrows). (For interpretation of the references to colour in this figure legend, the reader is referred to the web version of this article.)

Abdominal ultrasound showed hetero-echoic lesions on the posteroinferior of the bladder wall without hydronephrosis in both kidneys (Fig. 2). On CT-IVU (*CT-Intra Venous Urogram*) examination of the bladder, distal ureteral dilation was identified with a size of ±4.1 × 3.4 cm and a wall thickness of ±0.3 cm. This ureteral dilation showed fluid density which was filled with contrast in Delayed Phase fill. A stone with the size of ±0.9 × 0.6 cm was also identified on the left UVJ which did not cause obstruction (Fig. 3).

**Fig. 4:**
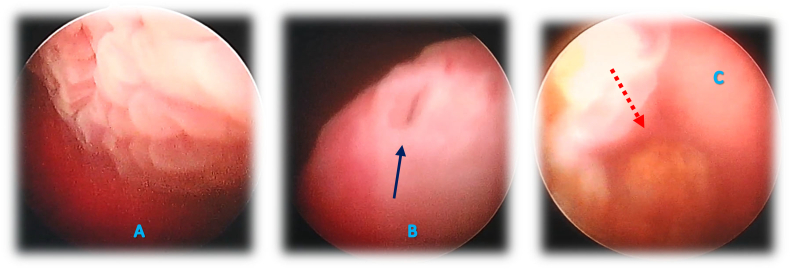
A left ureterocele saw during a cystoscopy (A). The blue arrow indicates the opening of the ureterocele (B). The red arrow indicates the left UVJ stone found during ureterocele resection (C).

The definitive management of this case is ureterocele resection surgery using endoscopy. As initial treatment, the reduction of prolapsed mass was performed manually by pushing the mass back into the urethra. Then, a cystoscopy was performed which identified the presence of left ureterocele (Fig. 4). We continued to perform the left ureterocele resection procedure using endoscopic resection. During this intraoperative procedure, a stone was found in the left UVJ (Fig. 4C). After completing the resection procedure, RPG was performed on the dilated ureter and it was found that the ureterocele connected to the upper moiety double system in the left kidney. The procedure run smoothly without any difficulties. One year following surgery, the patient was asymptomatic without the presence of renal function deterioration or urinary tract infection.

The results of the histopathology examination of the resected tissue revealed the tissue was partially coated by transitional epithelium and squamous epithelium. Infiltration of neutrophil inflammatory cells, lymphocytes, and bleeding areas was identified, and there were no signs of malignancy. The result concluded that the tissue was part of the ureterocele.

## 3 Discussion

Ureterocele is a sacculation or cystic dilatation of terminal ureter [7]. Ureterocele occurs in about 1 of every 4000 births where females are 4–7 times more likely to experience this condition compared to males [3]. In this study, we reported a rare case of ureterocele where the ureterocele began to appear in adulthood, with a clinical appearance of a vulvar mass. Ureterocele in adults is rarely diagnosed and is generally asymptomatic and usually results in a delay of treatment. In this case, the patient arrived on time, allowing for proper management and a speedy recovery.

In adolescents or young adults, the clinical symptoms that were usually reported in ureterocele are infection, abdominal pain, and incontinence [8]. Ureterocele that prolapsed out of the urethra is very rare and usually leads to acute urinary retention. In female, ureterocele is in a form of vulvar mass that can be reduced [9]. The incidence of prolapsed ureterocele is still unknown. One study stated that less than 5 % of all symptomatic ureterocele cases led to prolapsed mass that exits through the urethral meatus [10]. From several kinds of literature, the incidence of prolapsed ureterocele that appeared as vaginal protrusion is 5–10 % in all age groups and was found more commonly in newborns [11]. Prolapsed ureterocele is rare and more common in women mainly due to the shorter length of the urethra and weakness in the distal urethral wall [12].

There are several differential diagnoses of prolapsed vulvar mass besides ureteroceles such as paraurethral cysts, urethral prolapse, urethral polyps, or prolapsed pelvic organs which include cystocele, urethrocele, rectocele, and enterocele. The prolapsed pelvic organ is a condition where the female pelvic organ descends, including the bladder, uterus, vagina, and small and large intestines, which results in protrusion of the vagina, uterus, or both. This condition is caused by weakness of the muscles, fascia, and ligament supporting pelvic organs [13], [14].

In this patient, ultrasonography and CT IVU investigations were performed. This modality is used as the initial diagnostic tool for ureterocele or other prolapsed vulvar mass cases. Ultrasonography also provides an important role in prenatal screening. Ultrasonography could also be used when symptoms which were related to ureterocele are present [2]. Ultrasound examination is able to visualize the dilatation of the ureter associated with the upper pole duplicated kidney and the presence of ureterocele inside the bladder with proximal ureteral dilatation [15]. CT scans with and without intravenous contrast could be used to determine the size, shape, and location of the ureterocele. In addition, CT scans allow the identification of a double kidney system, ureteral contour, hydronephrosis rate, cortical thickness in each part, the functional ability of kidneys to excrete contrast material, and other urogenital anatomic anomalies. CT IVU revealed a complete pelvic-ureteral duplication (double system). The literature mentioned that 80 % of ureterocele cases are related to upper kidney moiety with a double system [16].

Based on CT IVU and during surgery, a stone was found in the left vesicoureteral junction. The stone was then entirely removed and no further treatment was required. One of the ureterocele complications is a ureteral stone with a fairly frequent incidence, according to the literature this particular condition occurs in 4–39 % of cases, and most of them are solitary stones formed by stagnant urine and/or chronic infections. When distal ureteral stones develop, these stones cannot spontaneously pass due to the obstruction in the ureterocele estuary.

Initial management of prolapsed ureterocele is intended for mass decompression. Manual reduction/repositioning of ureterocele into the bladder should be initially performed even though recurrence may occur [17]. Surgical treatment of ureterocele consisted of incision, multiple punctures, unroofing, or resection. Currently, endoscopic incisions are the definitive procedure for the management of ureterocele. However, this procedure may risk the subsequent second procedure due to redundant ureterocele. Endoscopic incision of ureteroceles may result in VUR and the incidence ranges from 0 to 32 % in some patients [18]. Considering the complex presentation in this case and to prevent the subsequent secondary procedure due to re-prolapsed, we decided to directly undergo endoscopic resection. Ureterocele management itself is still controversial between endoscopic decompression, partial nephroureterectomy, or complete primary reconstruction. The modality of ureterocele management depends on the following criteria: patient's clinical status (with/without urosepsis), patient's age, duplicated kidney upper pole function, presence of reflux, obstruction of the ipsilateral ureter, and contralateral ureteral pathology. The goals of therapy should be clearly defined and factored into the clinical decisions. Although specific and definitive treatments for ureterocele differ in certain individuals, the goals remain the same: securing kidney function; preventing infection, preventing obstruction and reflux, and maintaining proper urinary continence [3], [5].

Long-term follow-up to assess the renal function, symptoms and the presence of vesicoureteral reflux is required in these patients [15]. Patients with the duplex system were significantly more likely to require an additional operation due to re-prolapse compared to patients with a single system [19]. Therefore, close follow-up and monitoring should be undertaken in these types of patients. One year following surgery, the patient was asymptomatic without renal function deterioration or urinary tract infection. Monitoring of renal function also showed promising result.

## 4 Conclusion

Ureterocele is a rare urological disease that is often asymptomatic in the early stage. In this case, ureterocele appears with the initial symptom of prolapsed vulvar mass which is a very rare case of ureterocele and is usually found in women. Radiological findings using ultrasonography and CT-IVU revealed distal dilatation of the left ureter in the form of ureterocele accompanied by a complete duplex system and left UVJ stone. Ureterocele management ranged from either operative or non-operative procedures that aim to overcome the occurrence of vesicoureteral reflux. Endoscopic resection is performed in the management of this case to prevent the occurrence of re-prolapsed ureterocele.

## Informed consent

Informed consent has been obtained prior participation of the patient.

## Ethical approval

The approval for this case report has been given by the Ethic Committee of the Health Research of Dr. Soetomo General-Academic Hospital, Surabaya, East Java, Indonesia. Ethic number: 0024/129/V/2020.

## Funding

This research did not receive any specific grant from funding agencies in the public, commercial, or not-for-profit sectors.

## Author contribution

Lalu Muhammad Editia Subihardi: conceptualization, validation, writing – original drafting, writing – review & editing

Ilham Akbar Rahman: writing – original drafting, writing – review & editing

Niwanda Yogiswara: writing – original drafting, writing – review & editing

Fikri Rizaldi: Supervision, validation, writing – review & editing

Tarmono: Supervision, validation, writing – review & editing

## Guarantor

Fikri Rizaldi, Department of Urology, Faculty of Medicine, Universitas Airlangga, Universitas Airlangga Teaching Hospital, Surabaya, East Java, Indonesia

## Research registration number

1. Name of the registry: Not required
2. Unique identifying number or registration ID: Not required
3. Hyperlink to your specific registration (must be publicly accessible and will be checked): Not required

## Conflict of interest statement

The authors declare that there are no conflicts of interest.
